# Outdoor air pollution and hospitalizations for ischemic heart disease: a systematic review and meta-analysis

**DOI:** 10.3389/fpubh.2025.1643134

**Published:** 2025-08-05

**Authors:** Mailidan Maimaitiniyazi, Abuduwupuer Haibier, Muyesaier Maimaitiniyazi, Meiheliya Maisuti, Ailifeire Aihaiti, Tuersunayi Yisimiti, Muyesai Nijiati

**Affiliations:** ^1^Emergency Center, People’s Hospital of Xinjiang Uygur Autonomous Region, Urumqi, China; ^2^Xinjiang Medical University, Ürümqi, Xinjiang, China

**Keywords:** ischemic heart disease, hospitalization risk, meta-analysis, outdoor air pollutants, relative risk

## Abstract

**Objective:**

The study aimed to evaluate the association between six major outdoor air pollutants (i.e., PM_2.5_, PM_10_, NO_2_, SO_2_, CO, and O_3_) and the risk of ischemic heart disease (IHD)-attributable hospitalization to provide potential scientific evidence for the formulation of clinical and public health policies.

**Methods:**

Using the PubMed and Web of Science databases (up to March 2025), the present meta-analysis screened and included 17 high-quality studies (three cohort studies (CS), one case–control study (CC), and 13 time-series studies (TS)) covering 28,186,905 cases hospitalized for IHD. This study focused on the determination of the impact of air pollutant concentration changes on the risk of IHD-attributable hospitalization using statistical analysis in Stata 16.0. In addition, sensitivity analyses and funnel plots were employed to assess stability and publication bias.

**Results:**

The meta-analysis revealed the following associations between the air pollutants and IHD-attributable hospitalization: PM_2.5_: [RR = 1.01 (95% confidence interval (CI):1.00 ~ 1.01), *p* = 0.000], PM_10_: [RR = 1.01 (95%CI:1.00 ~ 1.01), *p* = 0.000], NO_2_: [RR = 1.02 (95%CI: 1.00 ~ 1.03), *p* = 0.000], SO_2_: [RR = 1.01 (95%CI: 1.00 ~ 1.02), *p* = 0.001], CO: [RR = 1.04 (95%CI: 0.97 ~ 1.12), *p* = 0.01], and O_3_: [RR = 1.00 (95%CI: 1.00 ~ 1.00),*p* = 0.000].

**Conclusion:**

High concentrations of air pollutants may significantly contribute to an increased risk of IHD-attributable hospitalization. The present study identifies air pollution as a major modifiable cardiovascular risk factor that should be integrated into clinical cardiovascular disease (CVD) management and public health policy formulation.

## 1 Introduction

Cardiovascular disease (CVD) is a leading cause of global mortality and excessive healthcare costs. Harmful health behaviors and environmental factors may have contributed to persistently elevated CVD mortality rates over the past three decades, despite the implementation of multifaceted prevention strategies and therapeutic advances (1). As a major manifestation of CVD, ischemic heart disease (IHD) develops due to coronary artery stenosis or occlusion, which may trigger myocardial ischemia, hypoxia, necrosis, and complications such as congestive heart failure and arrhythmias (2). Projections indicate that IHD will remain the primary cause of cardiovascular mortality by 2050 (3), underscoring the ongoing challenge in its prevention and treatment. The risk of IHD is modulated by genetics, age, sex, comorbidities, and environmental exposures, among which air pollution is a significant contributor (4). The World Health Organization (WHO) estimates that ambient air pollution causes 4.2 million premature deaths annually, with IHD and stroke accounting for approximately 68% of this attributable mortality (5). Common pollutants include particulate matter (PM), ozone (O_3_), carbon monoxide (CO), sulfur dioxide (SO_2_), and nitrogen oxide (NO) (4, 6, 7). All these pollutants may induce systemic inflammation, oxidative stress, endothelial dysfunction, hypercoagulability, and thrombosis, resulting in exacerbated risk of IHD (8). Consequently, reducing air pollution exposure is imperative for IHD prevention. Air pollution is a severe regional and global issue with profound impacts on economies, societies, tourism, and health (9). It is pivotal to investigate the role of outdoor air pollution exposure in IHD development, which may facilitate our in-depth understanding of the link between air pollution and CVD, thereby providing a scientific basis for formulating effective public health policies. There have been individual reports on this topic, with biased focus on single pollutants and limitations such as small sample sizes and significant methodological variations, leading to inconsistent results and difficulty in drawing definitive conclusions (10, 11). In view of the above interpretations, this study systematically evaluated the association between six major outdoor air pollutants (PM_2.5_, PM_10_, NO_2_, SO_2_, CO, and O_3_) and the risk of IHD-attributable hospitalization through a comprehensive literature synthesis and analysis. Through this investigation, it may provide additional evidence to understand the relationship between air pollution and CVD, underscore its importance as a modifiable risk factor, and provide targeted recommendations for clinical practice and public health policymakers, thereby preventing and controlling CVD effectively.

## 2 Materials and methods

### 2.1 Literature retrieval strategy

The literature search strategy was developed and implemented by the first author (the searcher). Through searches in PubMed and Web of Science primarily, literature retrieval was conducted based on search term combinations consisting of three parts: (1) "Air pollutants" OR "Particulate matter" OR "PM_2.5_" OR "PM_10_" OR "Nitrogen dioxide" OR "NO_2_ " OR "Sulfur dioxide" OR "SO_2_" OR "CO" OR "Ozone" OR "O_3_"; (2) "Ischemic heart disease" OR "IHD" OR "Coronary Heart Disease" OR "CHD"; and (3) "Patient admission" OR "Hospitalization" OR "Admission." The final search strategy combined these three sets of terms using the Boolean operator “AND”: (1) AND (2) AND (3). The search timeframe covered English-language articles published from the inception of each database up to March 2025.

### 2.2 Inclusion and exclusion criteria

#### 2.2.1 Inclusion criteria

1. Study population: The study population included patients with IHD, including those with coronary heart disease (CHD).
2. Study type: The study types included were time-series studies (TS), case–control studies (CC), or cohort studies (CS).
3. Exposure factors: The exposure factors included studies exploring the impact of outdoor air pollution (e.g., PM_2.5_, PM_10_, NO_2_, SO_2_, CO, O_3_, etc.) on IHD-attributable hospitalization, with only single-pollutant effects being included.
4. Outcome measures: The outcome measures required studies to report the risk of IHD-attributable hospitalization associated with air pollution [risk ratio (RR) and its 95% confidence interval (CI)].

#### 2.2.2 Exclusion criteria

The exclusion criteria were as follows: (1) Studies that did not clearly define the study population as patients with IHD; (2) studies that did not examine the association between air pollutants (PM_2.5_, PM_10_, NO_2_, SO_2_, CO, and O_3_) and IHD-attributable hospitalization; (3) studies reporting mixed pollutant effects without extractable data on single-pollutant impacts; (4) studies with incomplete original data; (5) duplicates; and (6) studies that did not provide or could not convert results into RR and its 95% CI.

### 2.3 Data extraction

The retrieved literature was first de-duplicated using EndNote. Then, two authors independently screened the titles and abstracts of the de-duplicated literature, excluding articles that were clearly irrelevant. Subsequently, our predefined inclusion and exclusion criteria were further referenced to implement a secondary screening, followed by a full-text review for further selection. Furthermore, regarding the final selected literature, quality assessment and data extraction were conducted by two researchers independently, followed by cross-checking and consolidation. In case of discrepancies, a third independent investigator was involved to review and verify the results. The final decision was made through discussion, and if necessary, the corresponding author was contacted to obtain complete raw data.

### 2.4 Quality assessment of the included literature

Among the 17 included studies (10–26), there were three cohort studies (10, 20, 26), one case–control study (22), and 13 time-series studies (11–19, 21, 23–25). The methodological quality of the included literature was evaluated using the Newcastle–Ottawa Scale (NOS) to identify the risk of bias across three domains: selection, comparability, and outcome. With a maximum score of 9, this scale classified studies scoring ≥7 as high-quality.

### 2.5 Statistical analysis

All statistical analyses were performed using the STATA 16.0 software. Effect measures were expressed as risk ratio and relative risk (RR) with 95% CI. Heterogeneity was assessed using the I^2^ statistic. A fixed effects model was applied in the presence of low heterogeneity [I^2^ < 50% (*p* > 0.1)], while a random effects model was used in the case of significant heterogeneity [I^2^ ≥ 50% (*p* ≤ 0.10)]. Sensitivity analyses were conducted to identify potential sources of heterogeneity. The sensitivity analyses were performed by sequentially excluding individual studies to examine their influence on the overall effect size. Forest plots were generated to present the meta-analysis results, and funnel plots were used to assess publication bias. The associations of IHD-attributable hospitalization rates with PM_2.5_, PM_10_, O_3_, SO_2_, and NO_2_ were analyzed per 10 μg/m^3^ increment, while the association with CO was assessed per 1 mg/m^3^ increment. A *p*-value < 0.05 was considered statistically significant.

## 3 Results

### 3.1 Literature search results and screening flowchart

**Figure 1:**
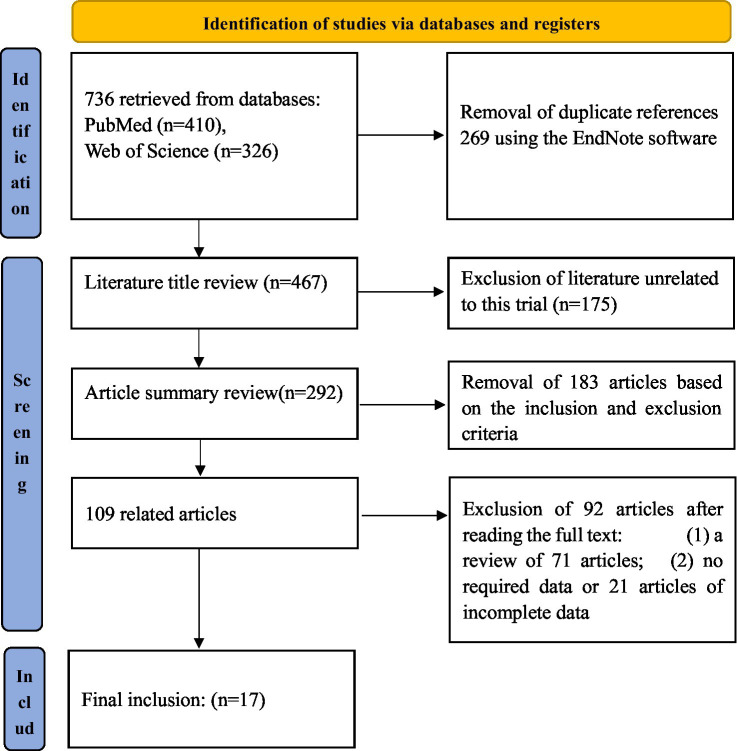
Literature screening flow chart.

An initial retrieval resulted in the inclusion of 736 records. Finally, 17 (10–26) studies were ultimately included in the meta-analysis following the removal of 269 duplicate articles, 175 irrelevant studies, 183 articles that did not meet the inclusion/exclusion criteria, 71 review articles, and 21 studies with missing or incomplete data. The total number of cases analyzed was 28, 186,905 (Figure 1). The baseline characteristics of the included studies are presented in Table 1.

**Table 1:** Baseline characteristics of the included studies.

Author	Study region	Study Design	Sample size (cases)	Sex (m/f)	Age	Pollutants	Follow-up periods	NOS grade
Liu et al. (12)	China (Lanzhou)	TS	88,805	59,507/29,298	<65 = 46,562 >65 = 42,243	PM_2.5_, PM_10_, NO_2_, SO_2_, CO, O_3_	2013–2020	8
Jiang, et al. (13)	China (Sichuan)	TS	104,779	55,891/48,888	<45 = 4,216 45–64 = 25,586 ≥65 = 74,977	PM_2.5_, PM_10_	2017–2018	8
Jiang et al. (10)	China (70 Chinese cities)	CS	6,444,441	/	/	O_3_	2015–2017	9
Feng et al. (14)	China (Anhui)	TS	16,656	9,967/6,689	<65 = 5,466 >65 = 11,190	NO_2_, O_3_, CO	2014–2021	7
Dąbrowiecki et al. (15)	Poland	TS	123,870	/	/	PM_2.5_, PM_10_, SO_2_, NO_2_	2012–2017	8
Xie et al. (16)	China (74 Chinese cities)	TS	2,670,000	/	<65 = 1,240,000 >65 = 1,430,000	PM_2.5_	2016–2017	7
Han et al. (17)	China (Zibo)	TS	21,105	/	/	PM_2.5_, PM_10_, O_3_	2015–2019	7
Zhang et al. (18)	China (Tianjin)	TS	15,570,000	/	/	PM_2.5_	2015–2017	8
Cao et al. (19)	China (Ganzhou)	TS	201,799	114,671/87,128	<65 = 99,921 >65 = 101,878	O_3_	2016–2020	8
Shamsa et al. (20)	US (Michigan)	CS	/	/	/	PM_2.5_	2010–2016	8
You et al. (21)	China (Ganzhou)	TS	201,799	114,671/87,128	<65 = 99,921 >65 = 101,878	PM_2.5_	2016–2020	9
Liu et al. (22)	China (20 provinces)	CC	387,817	/	/	PM_2.5_, PM_10_, SO_2_, NO_2_, O_3_	2013–2020	7
Dzhambov et al. (23)	Bulgaria	TS	98,567	/	/	PM_2.5_, PM_10_, SO_2_, NO_2_, CO, O_3_	2009–2018	9
Liu et al. (24)	China (Chengdu)	TS	33,017	/	/	PM_2.5_, PM_10_	2015–2016	8
Ban et al. (11)	China (Beijing)	TS	2,202,244	1,287,872/914,372	≤64 = 1,118,519 >64 = 1,083,725	PM_2.5_	2013–2017	9
Tam et al. (25)	China (Hong Kong)	TS	/	/	/	PM_2.5_, PM_10_, SO_2_, NO_2_, O_3_	2001–2010	8
von Klot et al. (26)	Five European Cities	CS	22,006	/	/	PM_10_	1992–2001	7

Design: “TS” = time-series studies, “CS” = cohort studies, and “CC” = case–control studies; NOS=Newcastle–Ottawa Scale.

### 3.2 Meta-analysis results

#### 3.2.1 PM_2.5_ and IHD-attributable hospitalization

**Figure 2:**
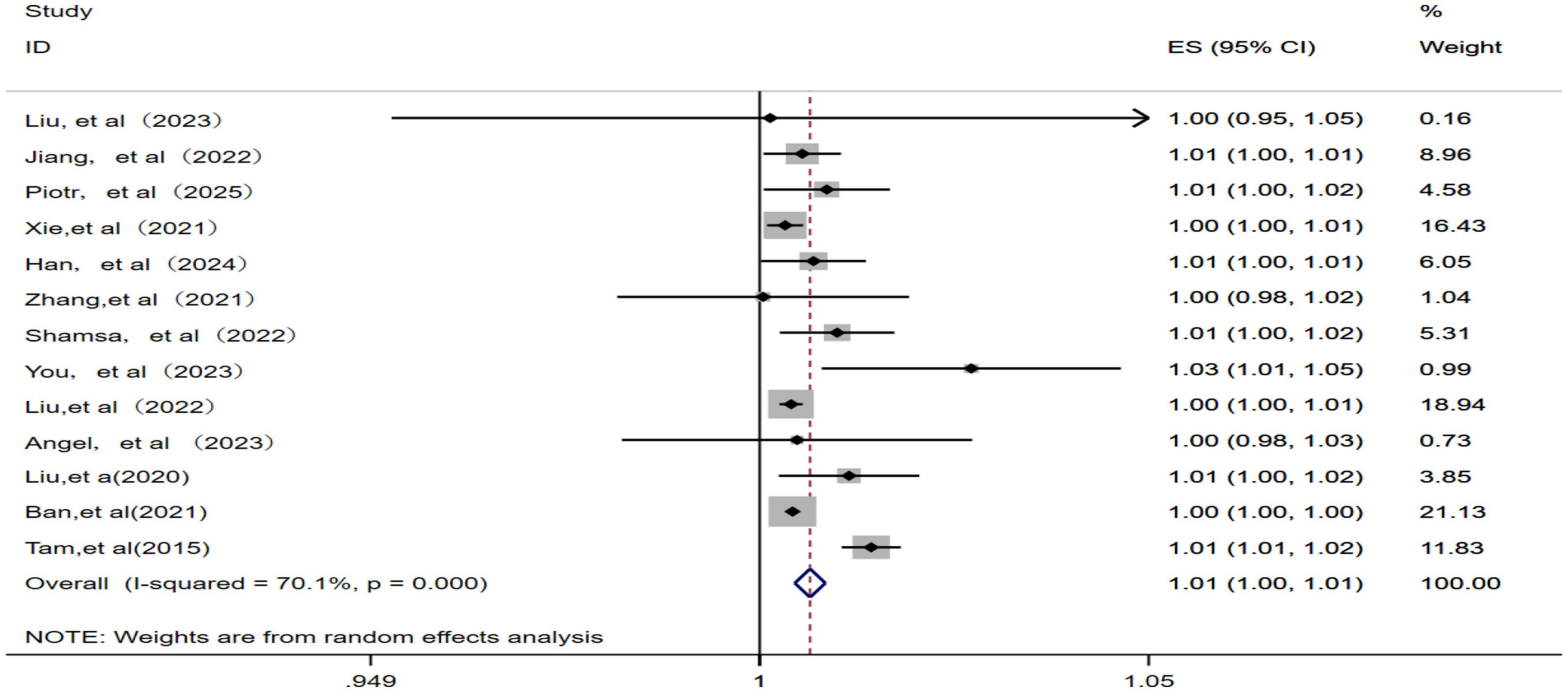
Forest plot of the meta-analysis on the association between PM_2.5_ exposure and IHD-attributable hospitalization.

Among the 13 studies (11–13, 15–18, 20–25) included, significant statistical heterogeneity was observed (*p* = 0.000, *I^2^* = 70.1%), resulting in the use of a random effects model. As a result, the pooled RR for IHD-attributable hospitalization was 1.01 (95% CI: 1.00–1.01) for every 10 μg/m^3^ increase in ambient PM_2.5_ concentration, as shown in Figure 2.

#### 3.2.2 PM_10_ and IHD-attributable hospitalization

**Figure 3:**
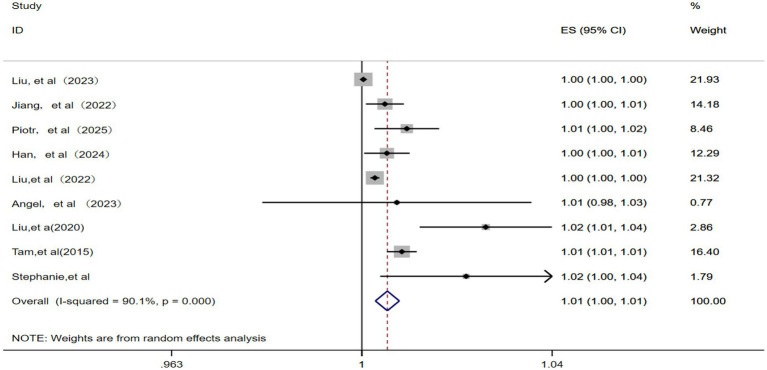
Forest plot of the meta-analysis on the association between PM_10_ exposure and IHD-attributable hospitalization.

This analysis included nine studies (12, 13, 15, 17, 22–26) and identified significant statistical heterogeneity (*p* = 0.000, *I^2^* = 90.1%). Therefore, a random effects model was applied. As illustrated in Figure 3, the pooled RR for IHD-attributable hospitalization was 1.01 (95% CI: 1.00–1.01) for every 10 μg/m^3^ increase in ambient PM_10_ concentration.

#### 3.2.3 NO_2_ and IHD-attributable hospitalization

**Figure 4:**
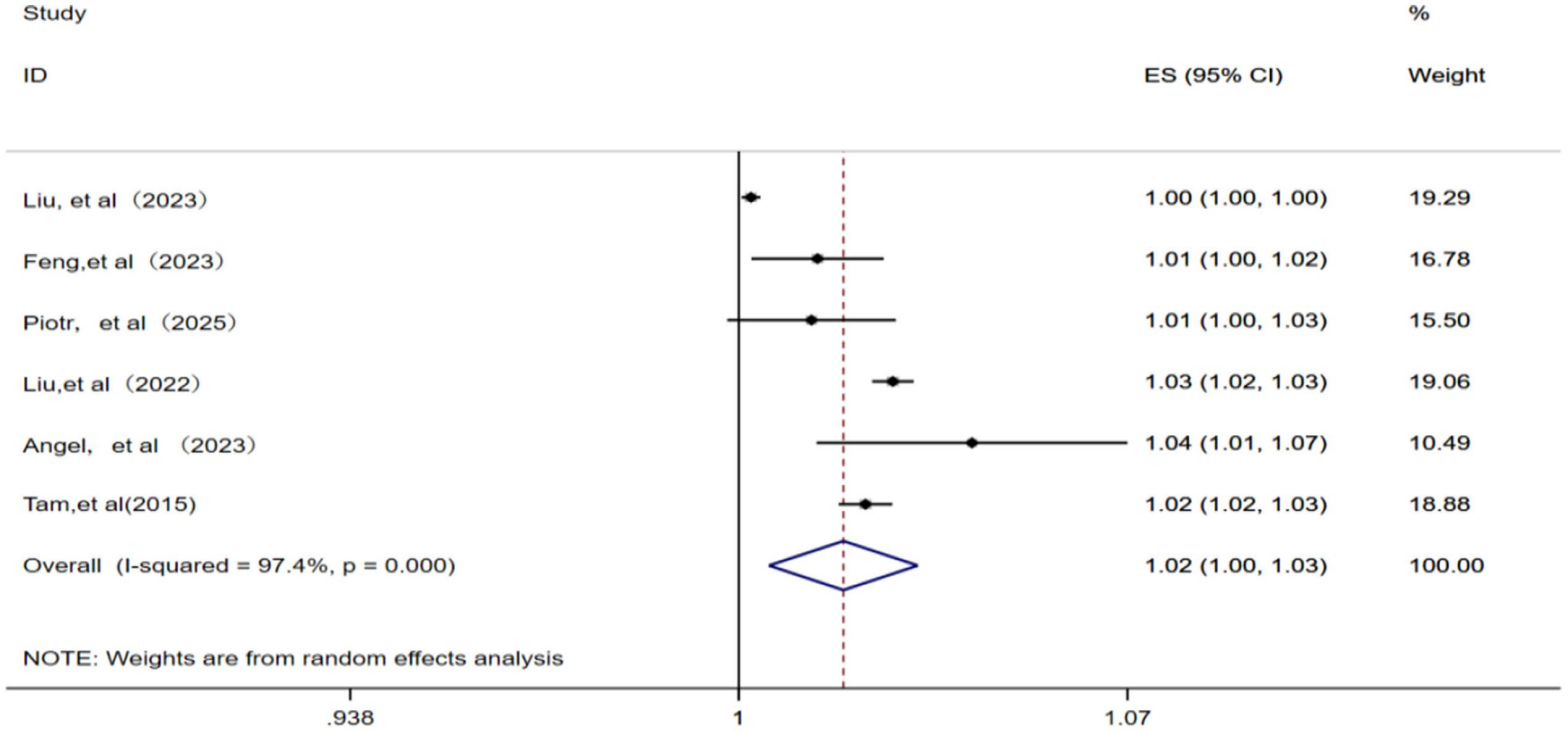
Forest plot of the meta-analysis on the association between NO_2_ exposure and IHD-attributable hospitalization.

A total of six studies (12, 14, 15, 22, 23, 25) were included for analyzing the relationship between NO_2_ and IHD-attributable hospitalization. A random effects model was applied given the presence of significant statistical heterogeneity among these studies (*p* = 0.000, *I^2^* = 97.4%). The meta-analysis (Figure 4) showed that the pooled RR for IHD-attributable hospitalization was 1.02 (95% CI: 1.00–1.03) for every 10 μg/m^3^ increase in ambient NO_2_ concentration.

#### 3.2.4 SO_2_ and IHD-attributable hospitalization

**Figure 5:**
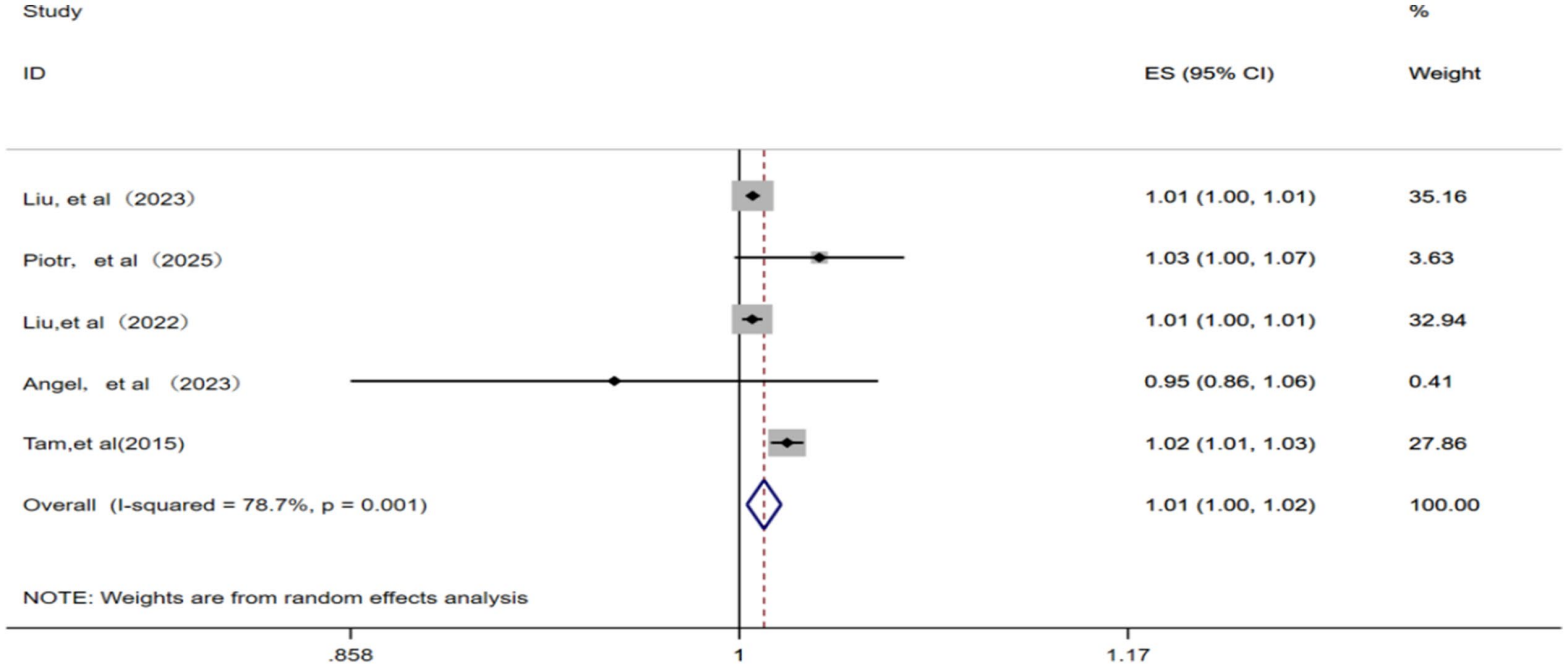
Forest plot of the meta-analysis on the association between SO_2_ exposure and IHD-attributable hospitalization.

A total of five studies (12, 15, 22, 23, 25) were included in the analysis. With the identification of significant statistical heterogeneity across the studies (*p* = 0.001, *I^2^* = 78.7%), a random effects model was applied for meta-analysis. Consequently, each 10 μg/m^3^ increase in ambient SO_2_ concentration was associated with a pooled RR of 1.01 (95% CI: 1.00–1.02) for IHD-attributable hospitalization, as depicted in Figure 5.

#### 3.2.5 CO and IHD-attributable hospitalization

**Figure 6:**
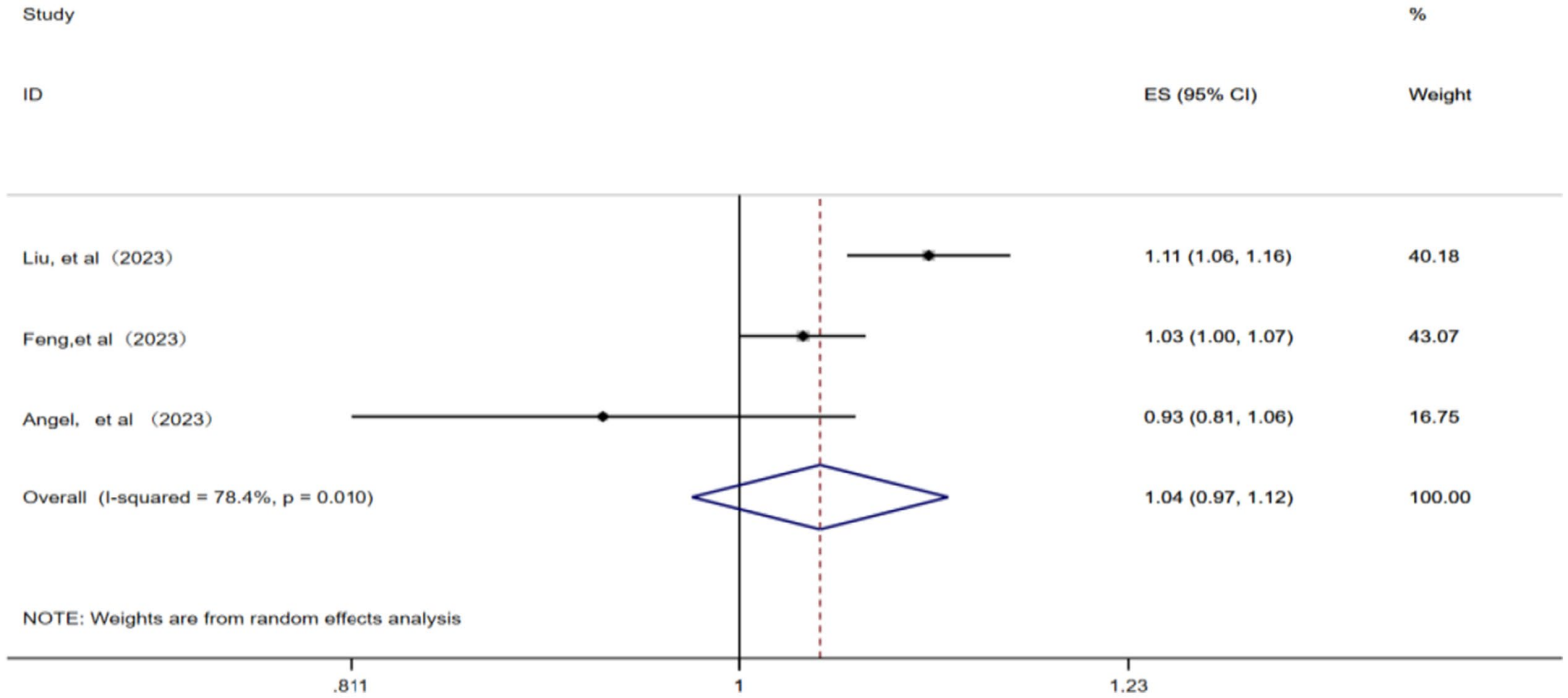
Forest plot of the meta-analysis on the association between CO exposure and IHD-attributable hospitalization.

A total of three studies (12, 14, 23) were included in this analysis. A random effects model was applied considering the presence of significant statistical heterogeneity among the studies (*p* = 0.01, *I^2^* = 78.4%). The meta-analysis revealed that for each 1 μg/m^3^ increase in ambient CO concentration, the pooled RR for IHD-attributable hospitalization was 1.04 (95% CI: 0.97–1.12), as illustrated in Figure 6.

#### 3.2.6 O_3_ and IHD-attributable hospitalization

**Figure 7:**
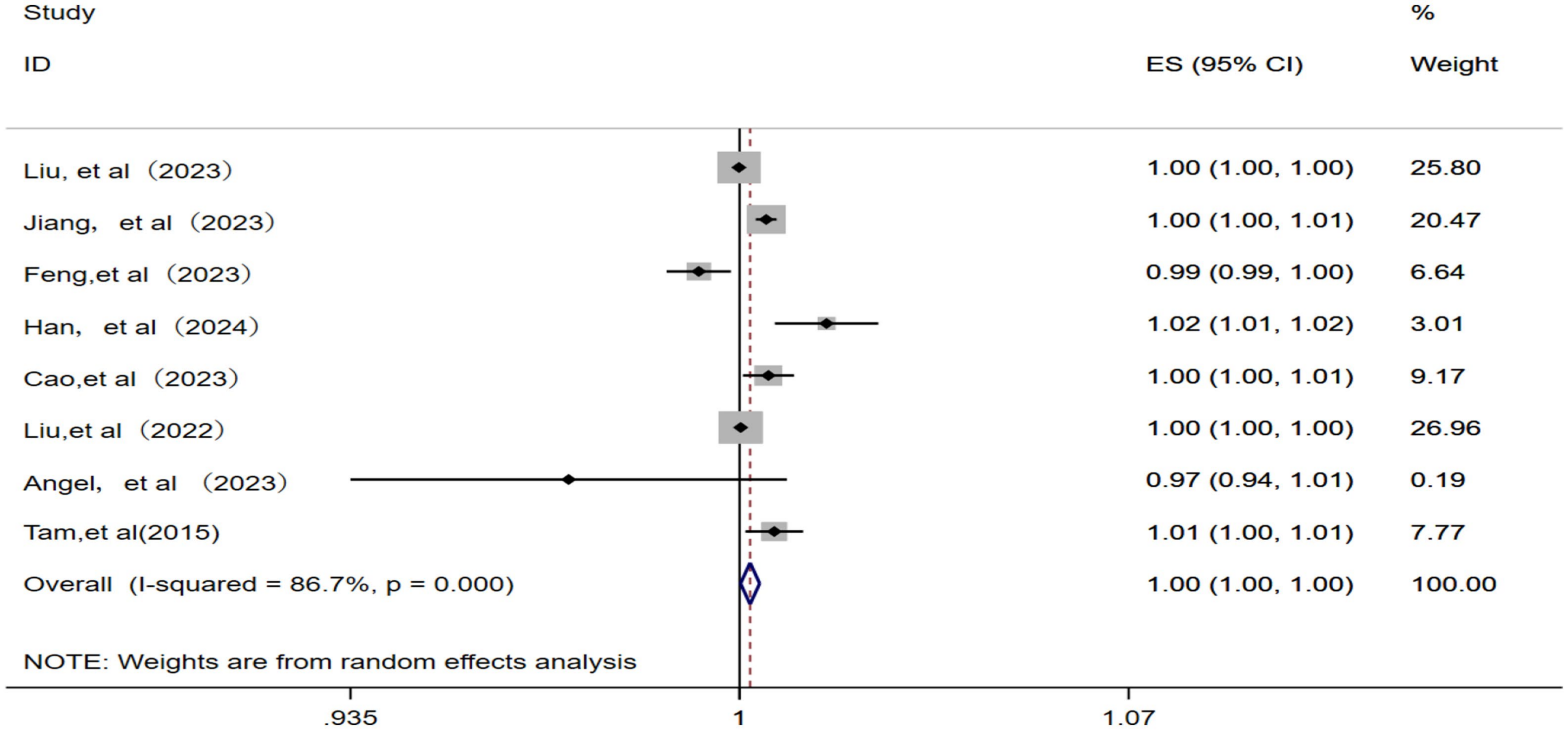
Forest plot of the meta-analysis on the association between O_3_ exposure and IHD-attributable hospitalization.

A total of eight studies (10, 12, 14, 17, 19, 22, 23, 25) were included for meta-analysis using a random effects model after the confirmation of significant statistical heterogeneity (*p* = 0.000, *I^2^* = 86.7%). The corresponding analysis (Figure 7) revealed that a 10 μg/m^3^ increase in ambient O_3_ concentration was associated with an RR of 1.00 (95% CI: 1.00–1.00) for IHD-attributable hospitalization, as presented in Figure 7.

## 4 Sensitivity analysis and publication bias

**Figure 8:**
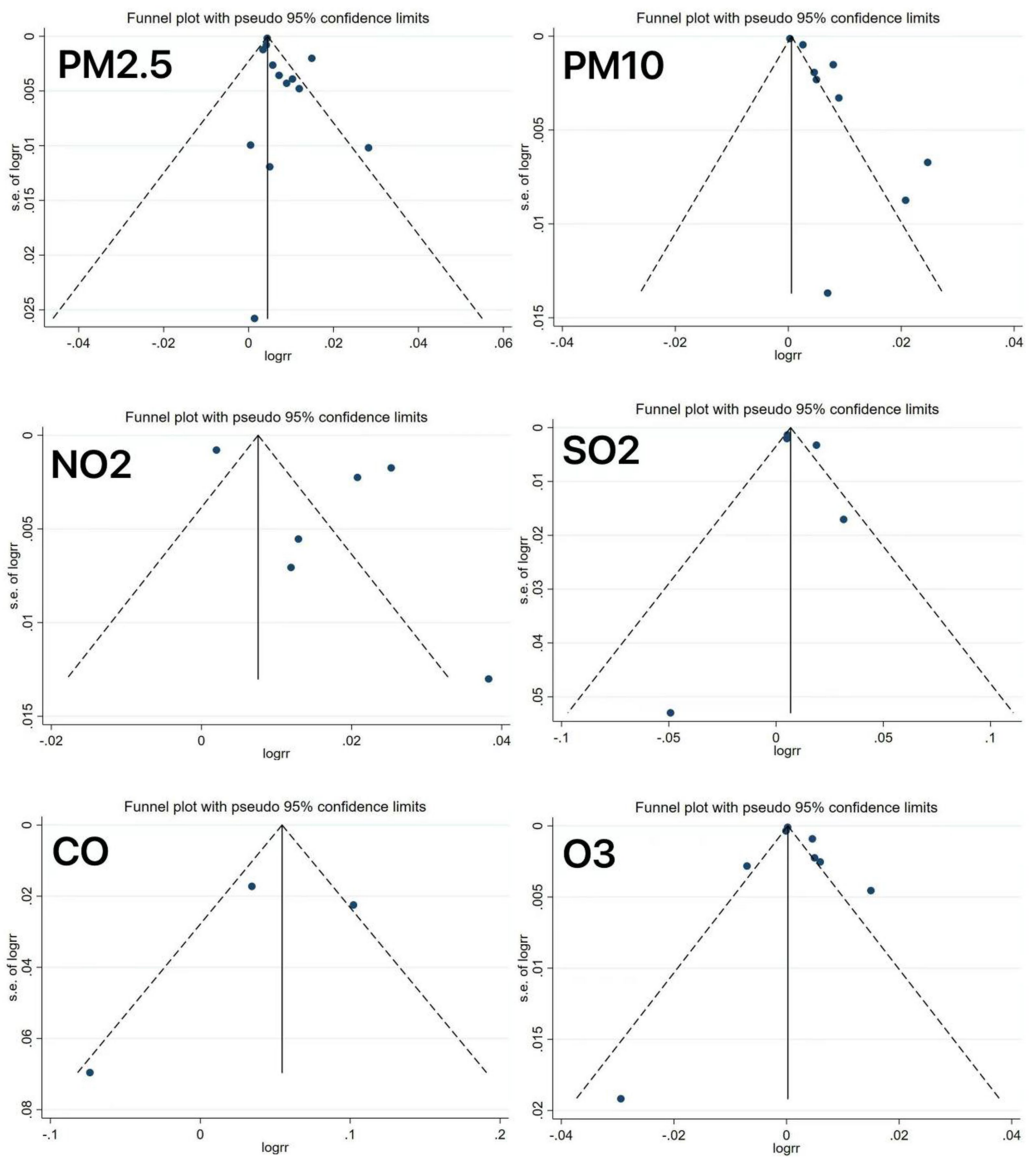
Funnel plots of the outcome indicators.

All meta-analyses showed significant heterogeneity across the outcomes. Following the sequential exclusion of the individual studies, the pooled effect estimates remained stable, with no substantial changes, indicating relatively robust results. As depicted in Figure 8, the funnel plot analysis revealed asymmetry in some studies, suggesting potential publication bias, which might be explained by the limited number of studies included.

## 5 Discussion

CVD remains the leading cause of death globally. The Global Burden of CVDs report revealed that the number of CVD-attributable deaths increased from 12.4 million in 1990 to 19.8 million in 2022. Among these, IHD had the highest age-standardized disability-adjusted life years among all diseases, at 2,275.9 per 100,000 population (1). In large prospective studies, traditional risk factors such as smoking, hypertension, dyslipidemia, physical inactivity, and diabetes have been confirmed as key etiological factors. Significantly, a more comprehensive understanding of these factors may greatly contribute to enhancing disease awareness, early detection, and the development of prevention and control strategies (27).

Air pollution is a significant yet often overlooked risk factor for CVD (28). In the context of lacking proactive intervention measures, CVD deaths are projected to double by 2050 compared to current levels. Most current epidemiological research focuses on the mortality risk of CVD associated with air pollution exposure, while there are relatively scarce studies on morbidity risk (e.g., hospitalization) (3). Therefore, the current study pooled effect estimates from 17 studies (10–26) for a meta-analysis to investigate the association between ambient air pollution exposure and IHD-attributable hospitalization rates in depth. As a result, our meta-analysis observed significant associations between long-term exposure to particulate matter (PM_2.5_ and PM_10_) and gaseous pollutants (O_3_, SO_2_, NO_2_, and CO) with increased IHD-attributable hospitalization rates (all *p* < 0.05).

A meta-analysis carried out by Pranata et al. (29) involving 84 cohort studies indicated that air pollutants (e.g., PM_2.5_, PM_10_, and NO_2_) could increase the risk of CVD. Inhaled PM initially triggers oxidative reactions in the lungs, subsequently amplifying into systemic vascular oxidative stress (30); subsequently, endothelial oxidative stress may deplete the availability of nitrites that regulate vascular tone and promote the progression of atherosclerosis by fostering pro-atherogenic factors and inhibiting anti-atherogenic factors. The impact of NO_2_ on disease is also similar to that of PM (31–33).

Numerous studies have linked gaseous pollutants (particularly ozone) to increased CVD incidence and hospitalization rates (6, 10, 34). Acute exposure to ambient O_3_ pollution—especially at levels exceeding the 8-h maximum concentration guideline of the WHO—demonstrates significant positive associations with hospitalization risks for major cardiovascular events, notably acute myocardial infarction and acute coronary syndrome. Both exposure levels below and above the WHO air quality guidelines were associated with excess hospitalization risks for cardiovascular events (10).

Moreover, additional common air pollutants, including SO_2_ and CO, originate primarily from fossil fuel combustion, industrial activities, and transportation. A study in Lisbon, Portugal, reported positive associations between CO, NO_2_, and SO_2_ levels and emergency admissions for cardiopulmonary diseases (35). In Boston, Massachusetts, pollutants (e.g., NO_2_, CO, and black carbon) also correlated positively with the rate of hospitalization for myocardial infarction (36). In addition, and consistently, two independent studies in Beijing, China, documented positive relationships between ambient air pollution (SO_2_, NO_2_, and PM_10_) and emergency admissions due to cardiovascular and respiratory causes (37, 38).

Climate change-induced global temperature rise creates photochemical conditions favoring pollutant formation, making them increasingly significant risk factors for health. The close connection between climate change and air quality indicates that long-term emission reductions to mitigate global warming may be a key factor in alleviating air pollution and improving air quality.

However, it should be acknowledged that this study still has several limitations. First, the included studies, despite high quality, could not fully account for confounding factors. Second, in addition to substantial heterogeneity, our sensitivity analyses and meta-regression failed to identify influencing factors, potentially due to the following reasons: (1) varied follow-up durations across the studies resulting from different study designs, which may have impacted the effect estimates and (2) inter-study differences in sampling methods, age and sex ranges, and sample sizes. In addition, funnel plot asymmetry in some analyses suggested potential publication bias, possibly leading to overestimation or underestimation of effects, which may compromise the reliability of the conclusion and misguide clinical decisions. Therefore, in the future, it is essential to conduct multi-center, large-sample studies to verify the specific roles of these factors in pollutant-specific emergency admission risks, along with stricter preregistration and improved transparency to reduce publication bias.

## 6 Conclusion

Our study confirms significant associations between six major ambient air pollutants—PM_2.5_, PM_10_, NO_2_, SO_2_, CO, and O_3_—and the risk of IHD-attributable hospitalization through a systematic review and meta-analysis involving 17 high-quality studies. The study clearly demonstrates that increased concentrations of these pollutants are significantly and positively correlated with the risk of IHD-attributable hospitalization. These findings may provide critical scientific evidence for clinical CVD management and public health policy formulation. Given that air pollution is a crucial, yet often neglected, modifiable risk factor in CVD, this opens an important avenue for prevention and control. Consequently, air pollution should be incorporated into CVD risk assessment and management systems by healthcare professionals clinically. Simultaneously, public health policymakers should focus on strengthening air quality monitoring and implementing effective improvement measures to tangibly mitigate the harm of air pollution to cardiovascular health in humans.

## Data Availability

The original contributions presented in the study are included in the article/supplementary material, further inquiries can be directed to the corresponding author.

## References

[1] Mensah GA Fuster V Murray CJL Roth GA Mensah GA Abate YH . Global burden of cardiovascular diseases and risks, 1990-2022. J Am Coll Cardiol. (2023) 82:2350–473. doi: 10.1016/j.jacc.2023.11.007, PMID: 38092509 PMC7615984

[2] Yazdanyar A Newman AB. The burden ofcardiovascular disease in the elderly: morbidity, mortality, and costs. Clin Geriatr Med. (2009) 25:563–77. doi: 10.1016/j.cger.2009.07.007, PMID: 19944261 PMC2797320

[3] Chong B Jayabaskaran J Jauhari SM Chan SP Goh R Kueh MTW . Global burden of cardiovascular diseases: projections from 2025 to 2050. Eur J Prev Cardiol. (2024) 13:zwae281. doi: 10.1093/eurjpc/zwae281, PMID: 39270739

[4] de Bont J Jaganathan S Dahlquist M Persson Å Stafoggia M Ljungman P. Ambient air pollution and cardiovascular diseases: an umbrella review of systematic reviews and meta-analyses. J Intern Med. (2022) 291:779–800. doi: 10.1111/joim.13467, PMID: 35138681 PMC9310863

[5] Sorensen C Lehmann E Holder C Hu J Krishnan A Münzel T . Reducing the health impacts of ambient air pollution. BMJ. (2022) 379:e069487. doi: 10.1136/bmj-2021-069487, PMID: 36223913

[6] Liang S Chen Y Sun X Dong X He G Pu Y . Long-term exposure to ambient ozone and cardiovascular diseases: evidence from two national cohort studies in China. J Adv Res. (2024) 62:165–73. doi: 10.1016/j.jare.2023.08.010, PMID: 37625570 PMC11331174

[7] Motesaddi Zarandi S Hadei M Hashemi SS Shahhosseini E Hopke PK Namvar Z . Efects of ambient air pollutants on hospital admissions and deaths for cardiovascular diseases: a time series analysis in Tehran. Environ Sci Pollut Res Int. (2022) 29:17997–8009. doi: 10.1007/s11356-021-17051-y, PMID: 34677770

[8] Rajagopalan S Al-Kindi SG Brook RD. Air pollution and cardiovascular disease: JACC state-of-the-art review. J Am Coll Cardiol. (2018) 72:2054–70. doi: 10.1016/j.jacc.2018.07.099, PMID: 30336830

[9] Sangkham S Phairuang W Sherchan SP Pansakun N Munkong N Sarndhong K . An update on adverse health efects from exposure to PM_2.5_. Environ Adv. (2024) 18:100603. doi: 10.1016/j.envadv.2024.100603

[10] Jiang Y Huang J Li G Wang W Wang K Wang J . Ozone pollution and hospital admissions for cardiovascular events. Eur Heart J. (2023) 44:1622–32. doi: 10.1093/eurheartj/ehad091, PMID: 36893798

[11] Ban J Ma R Zhang Y Li T. PM(2.5)-associated risk for cardiovascular hospital admission and related economic burdens in Beijing, China. Sci Total Environ. (2021) 799:149445. doi: 10.1016/j.scitotenv.2021.149445, PMID: 34365258

[12] Liu M Yu J Zhu A Ling J Chen R Zhang Y . Association between air pollution and coronary heart disease hospitalizations in Lanzhou City, 2013-2020: a time series analysis. J Urban Health. (2023) 100:1246–57. doi: 10.1007/s11524-023-00797-w, PMID: 38010484 PMC10728394

[13] Jiang W Chen H Liao J Yang X Yang B Zhang Y . The short-term efects and burden ofparticle air pollution on hospitalization for coronary heart disease: a time-stratified case-crossover study in Sichuan, China. Environ Health. (2022) 21:19. doi: 10.1186/s12940-022-00832-4, PMID: 35045878 PMC8767695

[14] Feng YT Lang CF Chen C Harry Asena M Fang Y Zhang RD . Association between air pollution exposure and coronary heart disease hospitalization in a humid sub-tropical region of China: a time-series study. Front Public Health. (2022) 10:1090443. doi: 10.3389/fpubh.2022.1090443, PMID: 36711381 PMC9874291

[15] Dąbrowiecki P Konduracka E Kołtowski Ł Chciałowski A Dąbrowiecka A Kępa P . Ambient air pollution and a risk of hospital admission due to acute and chronic coronary syndromes: a time-stratified case-crossover study in the 3 largest urban agglomerations in Poland. Pol Arch Intern Med. (2025) 135:16925. doi: 10.20452/pamw.16925, PMID: 39817670

[16] Xie Y Li Z Zhong H Feng XL Lu P Xu Z . Short-term ambient particulate air pollution and hospitalization expenditures of cause-specific cardiorespiratory diseases in China: a multicity analysis. Lancet Reg Health West Pac. (2021) 15:100232. doi: 10.1016/j.lanwpc.2021.100232, PMID: 34528013 PMC8342975

[17] Han C Cheng C Liu Y Fang Q Li C Cui F . Enhancing the health benefits of air quality improvement: a comparative study across diverse scenarios. Environ Sci Pollut Res Int. (2024) 31:44244–53. doi: 10.1007/s11356-024-33919-1, PMID: 38937357

[18] Zhang J Feng L Hou C Gu Q. Interactive efect between temperature and fine particulate matter on chronic disease hospital admissions in the urban area of Tianjin, China. Int J Environ Health Res. (2021) 31:75–84. doi: 10.1080/09603123.2019.1628928, PMID: 31190560

[19] Cao X You X Wang D Qiu W Guo Y Zhou M . Short-term efects of ambient ozone exposure on daily hospitalizations for circulatory diseases in Ganzhou, China: A time-series study. Chemosphere. (2023) 327:138513. doi: 10.1016/j.chemosphere.2023.138513, PMID: 36990357

[20] Shamsa EH Song Z Kim H Shamsa F Hazlett LD Zhang K. The links offline airborne particulate matter exposure to occurrence of cardiovascular and metabolic diseases in Michigan, USA. PLoS Glob Public Health. (2022) 2:e0000707. doi: 10.1371/journal.pgph.0000707, PMID: 36962575 PMC10021276

[21] You X Cao X Guo Y Wang D Qiu W Zhou C . Associations between short-term PM(2.5) exposure and daily hospital admissions for circulatory system diseases in Ganzhou, China: a time series study. Front Public Health. (2023) 11:1134516. doi: 10.3389/fpubh.2023.1134516, PMID: 36969639 PMC10034184

[22] Liu C Chen R Meng X Wang W Lei J Zhu Y . Criteria air pollutants and hospitalizations of a wide spectrum of cardiovascular diseases: a nationwide case-crossover study in China. Eco Environ Health. (2022) 1:204–11. doi: 10.1016/j.eehl.2022.10.002, PMID: 38077257 PMC10702887

[23] Dzhambov AM Dikova K Georgieva T Panev TI Mukhtarov P Dimitrova R. Short-term effects of air pollution on hospital admissions for cardiovascular diseases and diabetes mellitus in Sofia, Bulgaria (2009-2018). Arh Hig Rada Toksikol. (2023) 74:48–60. doi: 10.2478/aiht-2023-74-3704, PMID: 37014682 PMC10231894

[24] Liu S Wang L Zhou L Li W Pu X Jiang J . Differential efects of fine and coarse particulate matter on hospitalizations for ischemic heart disease: A population-based time-series analysis in southwestern China. Atmos Environ. (2020) 224:117366–6. doi: 10.1016/j.atmosenv.2020.117366

[25] Tam WWS Wong TW Wong AHS. Association between air pollution and daily mortality and hospital admission due to ischaemic heart diseases in Hong Kong. Atmos Environ. (2015) 120:360–8. doi: 10.1016/j.atmosenv.2015.08.068

[26] von Klot S Peters A Aalto P Bellander T Berglind N D'Ippoliti D . Ambient air pollution is associated with increased risk of hospital cardiac readmissions of myocardial infarction survivors in five European cities. Circulation. (2005) 112:3073–9. doi: 10.1161/CIRCULATIONAHA.105.548743, PMID: 16286602

[27] Gibbons GH Seidman CE Topol EJ. Conquering atherosclerotic cardiovascular disease - 50 years ofprogress. N Engl J Med. (2021) 384:785–8. doi: 10.1056/NEJMp2033115, PMID: 33657686

[28] Landrigan PJ Fuller R Acosta NJR Adeyi O Arnold R Basu N(N) . The lancet commission on pollution and health. Lancet. (2018) 391:462–512. doi: 10.1016/S0140-6736(17)32345-0, PMID: 29056410

[29] Pranata R Vania R Tondas AE Setianto B Santoso A. A time-to-event analysis on air pollutants with the risk of cardiovascular disease and mortality: A systematic review and meta-analysis of84 cohort studies. J Evid Based Med. (2020) 13:102–15. doi: 10.1111/jebm.12380, PMID: 32167232

[30] Bourdrel T Bind MA Béjot Y Morel O Argacha JF. Cardiovascular efects of air pollution. Arch Cardiovasc Dis. (2017) 110:634–42. doi: 10.1016/j.acvd.2017.05.003, PMID: 28735838 PMC5963518

[31] Suwa T Hogg JC Quinlan KB Ohgami A Vincent R van Eeden SF. Particulate air pollution induces progression of atherosclerosis. J Am Coll Cardiol. (2002) 39:935–42. doi: 10.1016/S0735-1097(02)01715-1 11897432

[32] Cao Y Long J Ji Y Chen G Shen Y Gong Y . Foam cell formation by particulate matter (PM) exposure: a review. Inhal Toxicol. (2016) 28:583–90. doi: 10.1080/08958378.2016.1236157, PMID: 27706953

[33] Miller MR Shaw CA Langrish JP. From particles to patients: oxidative stress and the cardiovascular effects of air pollution. Futur Cardiol. (2012) 8:577–602. doi: 10.2217/fca.12.43 22871197

[34] Lv X Shi W Yuan K Zhang Y Cao W Li C . Hourly air pollution exposure and emergency hospital admissions for stroke: A multicenter case-crossover study. Stroke. (2023) 54:3038–45. doi: 10.1161/STROKEAHA.123.044191, PMID: 37901948

[35] Alves CA Scotto MG Freitas MdC. Air pollution and emergency admissions for cardiorespiratory diseases in Lisbon (Portugal). Quim Nova. (2010) 33:337–44. doi: 10.1590/S0100-40422010000200020

[36] Zanobetti A Schwartz J. Air pollution and emergency admissions in Boston, MA. J Epidemiol Community Health. (2006) 60:890–5. doi: 10.1136/jech.2005.039834, PMID: 16973538 PMC2566060

[37] Zhang Y Wang SG Ma YX Shang KZ Cheng YF Li X . Association between ambient air pollution and hospital emergency admissions for respiratory and cardiovascular diseases in Beijing: a time series study. Biomed Environ Sci. (2015) 28:352–63. doi: 10.3967/bes2015.049, PMID: 26055562

[38] Ma Y Zhao Y Yang S Zhou J Xin J Wang S . Short-term effects of ambient air pollution on emergency room admissions due to cardiovascular causes in Beijing, China. Environ Pollut. (2017) 230:974–80. doi: 10.1016/j.envpol.2017.06.104, PMID: 28753900

