# Correction: Outdoor air pollution and hospitalizations for ischemic heart disease: a systematic review and meta-analysis

**DOI:** 10.3389/fpubh.2025.1712503

**Published:** 2025-10-17

**Authors:** Mailidan Maimaitiniyazi, Abuduwupuer Haibier, Muyesaier Maimaitiniyazi, Meiheliya Maisuti, Ailifeire Aihaiti, Tuersunayi Yisimiti, Muyesai Nijiati

**Affiliations:** ^1^Emergency Center, People's Hospital of Xinjiang Uygur Autonomous Region, Urumqi, China; ^2^Xinjiang Medical University, Ürümqi, Xinjiang, China

**Keywords:** ischemic heart disease, hospitalization risk, meta-analysis, outdoor air pollutants, relative risk

Affiliation 2. Xinjiang Medical University, Ürümqi, Xinjiang, China; was omitted from the paper. This affiliation has now been added for author(s) “Mailidan Maimaitiniyazi, Abuduwupuer Haibier, Muyesaier Maimaitiniyazi, Meiheliya Maisuti, Ailifeire Aihaiti and Tuersunayi Yisimiti”.

The original version of this article has been updated.

